# “The role of case management in HIV treatment adherence: HPTN 078”

**DOI:** 10.1007/s10461-022-03644-2

**Published:** 2022-04-01

**Authors:** Elizabeth E. Tolley, Erica L. Hamilton, Natalie Eley, Allysha C. Maragh-Bass, Eunice Okumu, Iván C. Balán, Theresa Gamble, Chris Beyrer, Robert Remien

**Affiliations:** 1 Behavioral, Epidemiological & Clinical Sciences, FHI 360, 359 Blackwell Street, 27701 Durham, North Carolina United States; 2 Science Facilitation, FHI 360, Durham, NC United States; 3 UNC Center for AIDS Research, University of North Carolina, Chapel Hill, NC United States; 4 Department of Behavioral Science and Social Medicine, Florida State University College of Medicine, Tallahassee, FL United States; 5 Department of Epidemiology, Bloomberg School of Public Health, Johns Hopkins University, Baltimore, MD United States; 6 Department of Psychiatry, Columbia University, New York, NY United States

**Keywords:** Motivational interviewing, Case management, HIV treatment, Adherence

## Abstract

Adherence to care and antiretroviral therapy is challenging, especially for people living with HIV (PLWH) with additional co-occurring risk factors. Case management interventions, including motivational interviewing (MI), show promise to improve HIV treatment adherence, but few studies have examined how such interventions are delivered to or experienced by PLWH who have been reengaged in care. We conducted qualitative interviews with six case managers and 110 PLWH exiting from a randomized study (HPTN 078) who received a MI-based case management intervention in addition to standard patient-navigation services, or standard services only. Our study provided greater insight into the main findings from HPTN 078, including an in-depth description of the multiple barriers to adherence faced by this largely “out-of-care” population, as well as a more nuanced understanding of the benefits and challenges of implementing MI. A blend of MI plus more intensive interventions may be needed for PLWH facing multiple structural barriers.

**Supplementary Information:**

The online version contains supplementary material available at 10.1007/s10461-022-03644-2.

## Introduction

For people living with HIV (PLWH), achieving and maintaining viral suppression (VS) is critically important to managing their own health and reducing the risk of transmitting HIV to others. Despite a growing armamentarium of antiretroviral therapies (ART) that promote viral suppression, an estimated 47% of PLWH struggle with HIV medication adherence (1). Among PLWH who have received an HIV diagnosis, an estimated 42% do not receive routine monitoring for CD4 or viral load testing (1). Racial and ethnic minorities living with HIV appear to have elevated risks of non-adherence and low retention in care (2).

Barriers to medical care visits and medication adherence are numerous and include individual risk factors such as mental health and substance use problems (3, 4), interpersonal factors such as intimate partner violence and family-related trauma (5–7), and broader social and structural issues such as stigma (8) or lack of access to housing, employment or health insurance (9). Recent research suggests that PLWH with multiple, co-occurring risk factors, referred to as “syndemics,” face the greatest challenges to medication adherence (10, 11). For example, a U.S. study of 390 sexual minority men living with HIV who had recently participated in one of two different interventions showed there was an additive effect of psychosocial conditions (e.g., childhood sexual abuse, post-traumatic stress disorder, anxiety disorders, depression and various substance use behaviors) on viral load (12). The study, which used a mediation modeling approach, found a significant and additive effect of syndemics on non-adherence to ART over time. They also found that baseline syndemics predicted the likelihood of viral non-suppression over a one-year follow-up, and that this was likely explained by the effect of these syndemic conditions on non-adherence.

Various strategies have been evaluated to address these individual, social, and structural barriers to adherence. Several studies have examined the effect of provider relationships on patients’ medication adherence. In a study of 2765 PLWH on ART living in four major U.S. cities, respondents who indicated more frequent interactions with providers who provided positive support (informational, emotional) had higher scores on medication self-efficacy, leading to higher medication adherence (13). Cultivating positive patient-provider relationships is proposed as especially important to engage traditionally disenfranchised groups, including African American PLWH and substance-using groups (14). Some studies suggest delivering HIV care through a case manager approach, in which a trained social worker works with a patient to develop a personalized plan (15). A systematic review of 239 studies that included motivational interviewing (MI) concluded that MI may be an effective strategy to assist medication adherence among PLWH who face mental health or substance use issues (16). A multi-tiered intervention (Heart to Heart) based on MI, peer-support groups and patient navigation with 95 Black and Latino PLWH not engaged in ART treatment, found significant improvements in viral suppression (17). Financial incentives (18, 19) have been offered to address structural issues. Systematic review of integrated interventions concluded that combined approaches produced some favorable outcomes (20). Despite evidence that interventions including personalized MI alone or in the context of multilevel interventions could promote improved adherence to HIV treatment, few studies have examined how such interventions are delivered within clinic settings or how they are experienced by PLWH who have been reengaged in care.

## METHODS

This study provides an in-depth examination of participants’ experiences with case management in the context of an intervention trial titled “Enhancing Recruitment, Linkage to Care and Treatment among HIV-Infected Men Who Have Sex With Men in the United States”.

### Overview of HPTN 078

HIV Prevention Trials Network (HPTN) 078 was a randomized trial to compare the effectiveness of a case management intervention versus standard of care (SOC) on viral suppression (VS) among gay and bisexual men with unsuppressed HIV. The study was conducted through four HPTN trial sites including University of Alabama-Birmingham; Fenway Clinic in Boston, Massachusetts; Johns Hopkins University, Baltimore, Maryland; and Ponce de Leon Clinic in Atlanta, Georgia. It employed respondent driven sampling, supplemented by direct recruitment, to recruit participants who had been disengaged from or had never linked to care. Participants randomized to the intervention arm were assigned to a study-specific case manager (SCM) who had been trained in MI techniques and provided tailored support for ART adherence. Participants in the SCM arm could also elect to receive automated or personally recorded motivational messages, visit and medication reminders via text, email or phone, choosing the frequency and content of provider sessions and automated/personalized content (21). Over the duration of the study, six SCMs with diverse demographic backgrounds and counseling experience were engaged, including two women and four men; three SCMs were Black while three were white. Some men identified as MSM. Most SCMs had a master’s degree in education or social work while two had bachelor’s degrees. Only two mentioned prior experience with motivational interviewing (MI). SCMs were trained in MI as part of the study intervention. This training consisted of a total of three days of in-person trainings as well as viewing MI demonstration videos (22). SCMs attended an initial half-day in-person training with 1.5 and 1-day refresher trainings by the same trainer approximately nine and 16 months into the study. (23). SCMs were also offered monthly group and one-on-one sessions with a member of the Motivational Interviewing Network of Trainers. Training sessions included practice sessions that were rated for MI fidelity using the MITI V4. Both SCM participants and those randomized to SOC had access to clinic-based case managers (CCMs), patient navigators or other patient services available through the clinic.

The study screened over 1300 individuals, of whom 902 were living with HIV; the study enrolled 144 of the 154 individuals who were virally unsuppressed. After one year, 91% of participants were still in follow-up and almost half of all participants (48%) had achieved viral suppression. Viral suppression did not differ by study arm (24). Qualitative exit interviews were conducted to better understand the role of the case management intervention and patients’ overall experiences with clinical care and medication adherence.

The local institutional review board for each study site approved the protocol prior to study implementation. Participants provided written informed consent prior to any study procedures. HPTN 078 is registered on ClinicalTrials.gov (NCT02663219).

### Qualitative Sub-Study Procedures

Between March 2018 and January 2019, we conducted phone-based semi-structured interviews with a total of 111 participants who had participated in HPTN 078 through one of the four sites. The time between the qualitative interview and the month 12 visit (at which their last viral load test was taken) varied widely (-1 month to + 10 months). The interviews were conducted by a team of five interviewers, generally one interviewer per site with a back-up interviewer, who had received training in qualitative interviewing. Although contact with participants was facilitated by each site, qualitative interviewers were not associated with the clinic. The topic guide explored participants’ experience with HIV care as part of the study and more generally, challenges and facilitators of medication and care adherence, and perspectives on overall and sexual health. As part of the main protocol, participants provided written consent to be contacted at exit for the qualitative interview. Prior to beginning an interview, the former HPTN 078 participant reconfirmed verbal (audio-recorded) informed consent. In addition, telephone interviews were conducted with six SCMs to explore their own experiences, including their perspectives on the use of motivational interviewing and major barriers and facilitators of adherence encountered by study participants.

All interviews were audio-recorded, transcribed verbatim and typed into word documents. An initial team of seven coders used a team-based coding approach to first apply structural coding (e.g., coding by sequential questions in the topic guide) and then develop and apply a thematic codebook to transcripts in NVivo 12, a qualitative software program used to manage analysis of textual (and other) data. Thematic codes included current clinic support, study SCM interactions, non-Study CM interactions, housing, finances, transportation, substance abuse, mental health and HIV medication and care adherence. Team members could apply multiple codes to the same excerpt. Team members coded the first several transcripts independently and met to discuss and reconcile differences in how they applied the coding scheme. Subsequently, each team member was assigned a set of transcripts to complete with periodic coding of a shared transcript to check that coders were still applying the codebook in a similar manner. Over the course of six months, the team held three structural inter-rater coding reliability (ICR) meetings, one content focused ICR and one final structural + content ICR meeting.

Working with the previously coded data, this subsequent analysis was conducted by two team members to explore whether and/or how the support delivered by the SCMs and experienced by participants in the SCM arm differed from those in the SOC arm. Analyst 1 read coding reports related to CM interactions and developed memos by arm and VS status. Both analysts explored and completed a matrix, organized by arm and VS status, summarizing participant reports of secondary codes that emerged from initial investigations. These included specific experiences related to housing, mental health issues, religion and other personal or contextual information. Analyst 2 reviewed and developed memos related to the SCM interviews. The team specifically examined how study CMs and participants described their case management experiences and assessed whether and/or how the content and strategies used by study and non-study CMs differed. We also examined whether or how services desired and/or provided by case managers varied by VS status and study arm.

## RESULTS

*Sociodemographic characteristics* Most participants (88%) in the qualitative sub-study were Black or African American men between the ages of 22–50. Most (79%) self-identified as gay or homosexual.

**Table 1:** Baseline socio-demographics of qualitative sample by study arm and viral suppression status at study exit (N = 110)^a^

	SCM Arm (n = 56)		SOC Arm (n = 54)^a^		Total (n = 110)^a^	
	VS (n = 27)	Not VS (n = 29)	VS (n = 32)	Not VS (n = 22)	VS (n = 59)	Not VS (n = 51)
**Age** (median, range)	40 (19–60)	38 (19–62)	45 (20–60)	40.5 (20–61)	44 (19–60)	40 (19–62)
**Race** ^**b**^ Black or African American (n = 97) White (n = 11)						
	24	26	27	20	51	46
	3	1	5	2	8	3
**Hispanic origin** (n = 5)	0	3	2	0	2	3
**Education** High school diploma or less (n = 39) Vocational/trade/technical (n = 8) Some college, including AA (n = 49) BA/BS or higher (n = 14)						
	11	9	10	9	21	18
	1	2	4	1	5	3
	13	14	13	9	26	23
	1	5	5	3	6	8
**Employment** Not employed (n = 75) Part-time (n = 17) Full-time (18)						
	17	19	22	17	39	36
	6	5	3	3	9	8
	4	5	7	2	11	7
**Partner status** Single/Divorced/Widowed (n = 92) Having primary/main partner, not living together (n = 2) Living w/main partner (n = 14) Married/Civil union/Legal partner (n = 2)						
	22	26	25	19	47	45
	1	0	0	1	1	1
	4	2	6	2	10	4
	0	1	1	0	1	1
**Living situation** ^**c**^ Does not have a stable home (n = 10) Lives w/relatives (n = 30) Lives w/roommate(s) (n = 21) Lives w/partner/spouse (n = 16) Lives by self (n = 31)						
	2	2	2	4	4	6
	7	7	9	7	16	14
	4	8	5	4	9	12
	4	2	7	3	11	5
	10	9	9	3	19	12
**Sites** Birmingham, Alabama (n = 25^a^) Atlanta, Georgia (n = 35) Baltimore, Maryland (n = 33) Boston, Massachusetts (n = 17)						
	6	6	9	4	15	10
	6	13	8	8	14	21
	11	6	9	7	20	13
	4	4	6	3	10	7

^*a*^ *The Birmingham site also interviewed one participant who had an unknown VS status at the 12-month follow-up visit. Therefore, the total interviewed participants at the Birmingham site was n = 26. This participant with unknown VS status was not included in analysis of the total 111 interviewed, because VS status was missing; therefore, the total number in this table is 110*

^*b*^*Two* (2) *participants identified as bi-racial, thus Race categories sum to more than (N = 110)*

^*c*^ *Other=2 (one in SCM arm and one in SOC arm)*

As shown in Table 1, similar proportions of participants in the SOC and SCM arms were virally suppressed in our qualitative sample, as was found in the main study (24)

Despite similar proportions of participants by arm who were and were not virally unsuppressed at exit, our qualitative analysis identified important differences in how participants experienced interactions with their case managers, as well as the kinds of support that were needed and provided by study arm and by viral suppression status. In the remainder of this section, we describe these differences in greater detail, highlighting both participant and SCM perspectives. Additional illustrative quotes are presented in Table 2 (supplement), organized by study arm, general theme, and viral suppression status.

### Participants’ positive experiences with intervention arm SCMs

Almost half of **virally suppressed participants in the SCM arm** (n = 12 of 27) described their SCM in very positive terms, often emphasizing the relational aspects of his/her support. Some participants used the phrase “*like I was talking to a friend,”* while others compared their relationship to that of a family member. They described discussions with the SCM as being two-way, non-judgmental.

*Well, when they talked to me, they made it so like that I was talking to a friend and someone that I can trust. So, they made it easier to open up and just relax and be able to put everything on the table, without worrying that I was being judged or anything… Because I ended up meeting, I met someone and started a new relationship. And then, for them just to inquire about it and I was filling them in, and they could see the happiness in my face, and they kept encouraging me, knowing that it was going to be okay. That impressed me also. (50-year old Black participant at the Baltimore site)*

*She would talk to me like she was my mom. She gave me straight talk. She didn’t take no shit. You know? (60 year-old Black participant at the Birmingham site.)*

Most other virally suppressed participants in the intervention arm (11 of 27) said that they were *already in care*, and therefore did not take much advantage of the program. A few such participants suggested they enjoyed interacting with the SCM despite having no direct need for assistance. Others implied that they did not have much contact with the SCM, while adding that there was *“nothing to improve”* or clarifying that the study intervention was unnecessary for them personally, because they already had the disease under control.

*I mean in the past we haven’t had conversations like that. But ah, like I said, it’s always been, for me, it’s been my self-motivation that keeps me focused and, and, and has um kept me doing, what I had to do as far as taking medication to keep my health um in check. Um, as far as him helping me, not that much. And that’s not a, not a bad thing. I mean, […] If you’re saying if I benefitted from his help, then, no. But not in a bad way. (28 year-old Black participant at the Atlanta site)*

Only two of the virally suppressed participants in the SCM arm shared any concerns about their interactions with the SCM, both from the same clinic. One person described the SCM as being “*50/50*” and not really coming through with his need for an apartment. The other implied that the *“one-on-one”* nature of SCM counseling sessions felt *“harsh”* and *“very serious”.*

*I think that more people will feel more comfortable with a group instead of it being more one-on-one. Because when it’s one-on-one, it’s - it’s very serious. I’m not saying that, you know, HIV medication and discussions aren’t - shouldn’t be taken seriously, but it should be - it shouldn’t be so harsh on people because some people might have, um, a harder time coming to terms of what they - what - what they’re doing. (23 year-old Black participant at the Atlanta site.)*

**Unsuppressed intervention participants** were even more likely than those who were suppressed to describe SCMs in positive terms (n = 22 of 29). Like those who were virally suppressed, they used familiar terms *“like a friend”* or *“like a brother”.* Similarly, they emphasized the non-judgmental, caring relationships provided by SCMs - someone who really cared for them and who was rooting for their success. Indeed, several virally unsuppressed participants in the intervention arm regretted that they would no longer be seeing their SCM given the end of the study. For some, having developed a relationship over the course of the study, it was as if they were losing a friend or a family member.

*I’m ending the study, but you know, I was saying to [SCM1 name] the other day, it’s been so nice dealing with both of you and [SCM2 name]’s mother that passed away. I wrote him a card and got him a little gift, because I’ve got a friend who is an artist, and he does glasswork. So, … you know, kind of, we’ve developed some bonds in the time I’ve been in the study. Both gentlemen are so nice, [names of SCM1 and 2]. So, like I said, it’s bittersweet um you know, I didn’t want to participate at first, you know, it got kind of cumbersome keeping up with the appointments and the interviews. But then, I began to enjoy it. (55 year-old Black participant at the Boston site.)*

The remainder of these participants (n = 7) were somewhat more neutral about their SCMs, although none expressed any direct criticisms. They acknowledged some functional support provided by the SCM but did not indicate a more personal connection with their assigned SCM.

### Experiences with non-study CCMs depend on viral status

Like those in the intervention arm, half (n = 17 of 32) of **virally suppressed participants in the SOC arm** described their CCM in overtly positive terms. Some, for example, described their CCM as someone who truly loved his/her work and went beyond expectations, while others described their relationships with CCMs in more professional terms, identifying the CCM’s provision of specific services as well as the emotional support and non-judgmental approach that their CCM provided. None of the SOC arm participants, however, used terms like *“friend”* or *“family”*.

*He’s just an amazing guy. He’s just, the way he, he – you could tell that he really has a passion for it, he’s not here just because of his paycheck… (He) is the reason why I come there, come here. Uh he actually was but they, they switched, like they got – he actually uh, got promoted here, actually, but they – yeah, they kind of switched different social workers sometimes… Oh, the past year, it’s over the past year, yeah, that’s my, my new case worker. And she’s like, she has um, a passion for it as well. (29 year-old Black participant at the Birmingham site)*

About a third of virally suppressed participants in the SOC arm (n = 10) did not appear to have relationship with a case manager or patient navigator. Some directly denied they were ever assigned a CCM (n = 4). Others recollected having met with a CCM on occasion but noted that such people often turned over (n = 6). Typical responses included that *“the case manager has changed quite a bit,” “my case manager has changed three or four times”* or *“they switch your social worker around without you knowing”.*

A small number of virally suppressed participants described their non-study CCM encounters in negative terms (n = 4). Mostly, these few participants suggested that their CCMs did not proactively check in with them, were not responsive to their needs, and that it might take them *“forever to respond back to emails or phone calls.”*

*And she doesn’t seem like she’s personable. She’s like, you know, I mean, come on. Like, I’m gay and usually females are always like, “Hey,” well it’s like a good time. But with her, …it’s like we don’t click at all. And my lover has a social worker that’s on her job, does her thing, if she can’t get in contact with him on the phone, she sends an e-mail and lets him know. I mean, like she cares, and mine just don’t do that. So, I deal with her as little as possible. (33 year-old Black participant at the Birmingham site)*

In contrast, far fewer **unsuppressed participants in the SOC arm** (n = 6 of 22) developed a strong personal bond with their CCM. While some CCMs were described as “*very helpful,” “comfortable”* or even as *“going above and beyond,”* more unsuppressed SOC participants were either neutral (n = 11) or critical in their descriptions of CMs (n = 4). For example, some participants depicted the CCM as *“very casual”* or *“very tolerated”* but also stated s/he was just *“doing her job.”* Several emphasized the transience of staff, and at times, the poor treatment they had received from CCMs.

*I could tell, she was of no help to me at all. There were other case managers there that I would see in the hallway, and I would ask them the questions, and then of course they would refer me back to my case manager, who wouldn’t keep appointments, so on and so forth. (49 year-old White participant at the Atlanta site)*

Half of participants in the intervention arm (n = 28 of 56) also had concurrent interactions with non-study CCMs. Whether virally suppressed or not, their experiences with these staff were reflective of participants in the SOC arm. For example, more than half of intervention arm participants (18 of 28) also described their non-study CCMs in very positive terms, portraying these staff as respectful, caring, and dedicated professionals who provided valuable services. Like some in the SOC arm, however, others noted that caseworkers frequently “*moved on,” “might not remember their name,”* or were not responsive to calls and emails.

### Role of CMs (both intervention and SOC) among virally suppressed participants

Among **VS intervention participants**, the SCM’s role appeared to focus on addressing participant questions about medical issues, assisting them to make and keep their clinic visits and to stay on track with their medications and with other life goals. Some participants highlighted the important role that the SCM played in helping them confront emotional challenges and dealing with mental health issues. The non-judgmental approach was *like being in a therapy session*.

*…as far as like the um the study that I was going through with [de-identified], those, the case workers there, they allowed me to actually talk and open up and they listened to where I was able to, you know, express myself and get some stressful things off my chest. They were very helpful… they helped me get into contact with the people at [de-identified] that I needed to get in touch with, and as I started going through some of the clinics and they started seeing if I needed to see any other ones like mental health and all, others started opening up doors for other appointments for me. (45 year-old Black participant at the Baltimore site) - SCM arm*.

SCMs validated this idea. As one SCM (#2) emphasized, many of the participants were dealing with complex life issues. *“A lot of sessions became counseling sessions. Some serious drug use and mental health issues came up. Everything came out.”*

In contrast, **VS participants in the SOC arm** (n = 16 of 32) were more likely to describe getting support for insurance and/or keeping their appointments than they were to receive emotional support. Non-study CCMs helped participants fill out insurance claims, apply for Medicaid or the Ryan White program, process disability claims. Like their SOC counterparts, about a third of virally suppressed participants in the intervention arm also attributed support with appointment reminders and insurance to non-study CMs.

Some virally suppressed participants in both arms described having needs outside of the HIV services. These included requiring assistance with housing (8 in the SOC and 9 in SCM arms), linkages to mental health services (2 in SOC and 3 in SCM arms), access to food pantries or fitness programs (3 in SOC and 3 in CM arms), and transportation services (4 in SOC and 3 in SCM arms). Although SCMs provided these linkages at times, they were more often provided by non-study CCMs or other groups. In general, at the time of the exit interview, VS participants in both arms indicated that these non-HIV needs had been or were in the process of being met.

*Um, I have a great relationship with my care manager, because she helps me get a lot of things done in case I need um, if I have problems with housing, she gets me connected with the right person in housing. Um, she also helps me with getting co-pays and um anything I have to deal with my disability, trying to file for disability, that’s who I go to get the help that I need. So, she’s pretty much a handy person when it comes to me needing things. (48 year-old Black participant at the Baltimore site) – SCM arm*.

*Getting out of drugs. Okay.* And how did she help with that? *She stayed on my ass and got me a self-program… It’s something that we were working on now. Just sort of working on it, but I don’t know (how to) tie this one to that. But I’m – in my opinion, I think it’s working out great. (37 year-old Black participant at the Birmingham site) - SOC arm*.

Virally suppressed participants often expressed, however, that they also relied on other sources of support. Some point to their mothers or their children as their *major motivator.* Others draw on their faith in God or their own internal strength and experience to stay healthy and adherent. One participant, who described himself as living to serve others, both professionally as someone engaged in customer service and outside of work as a *praise and worship minister at a church*, explained his commitment to keeping his appointments like this:

*This is imperative with me. This is almost like a lifeline with me. Okay? It’s almost – needed. It’s just as imperative to me as eating, resting, driving, working… because first of all, I love my life. I love living. I love living wholesome, as opposed to partial. I’m not seeking for any type of breakdown during this entire journey. (53 year-old Black participant at the Birmingham site.) – SCM arm*.

Another, who was diagnosed with HIV in 1996 while in prison, struggled with active addiction and feelings of depression before accepting the help of various case managers and peer support groups to first accept his situation and then apply himself to improving it. He now looks towards using his inner strength to help others:

*According to what they told me, my viral load is 100 or less, which is good. Meaning that I’ve come a long, long way, thank God! And, if I keep taking my medicine, that I could be undetectable. That’s what I’m striving for, to be a peer support, um, coordinator. But, you have to be undetectable, so that’s something else. […] (My intentions are) Continue taking my medicine and just allowing God to strengthen me where I may be weak. (46 year-old Black participant from Baltimore) - SOC arm*.

### Challenges with care for virally unsuppressed participants

Half of unsuppressed participants in the intervention arm (14 of 29) described the SCMs’ efforts to follow up on missed visits, keep them informed about test results, and to *gently admonish, encourage* or get them to *commit to* their medication adherence. In general, participants perceived these efforts as showing how much the SCM cared about them as a person, not just a patient. At the same time, several participants acknowledged that “*you can’t twist a grown man’s hand.”* An Atlanta participant who suffered a relapse during the study described the following encounter when he came in for his next blood draw:

*He (SCM) said, “What’s going on?” And I broke down and he told me, he’s like, “You’ve got to change. You cannot stay out there on drugs. you’ve got to like commit.” You know he gave me the encouragement and the uh -- the showing that he really, really -- that someone cares about me --to get my life back together. He -- you know he -- it wasn’t fake. […] He wasn’t rushing to do the blood draw and things like that. He was really not rushing -- he wasn’t rushing. He wanted to hear what my pain and my problems were, and some people really don’t you know -- they’re all about just business […] I just, I let him know what -- that I had relapsed you know. His main issue was trying to get me back on my meds. That was his main issue coz that’s the only issue I let him really know that I needed. Everything else – I kept that hidden. (51 year-old Black participant at the Atlanta site) - SCM arm*.

Indeed, more than half of unsuppressed participants, regardless of study arm, described dealing with on-going non-HIV related challenges – issues that were not always addressed by a case manager. These larger life problems, including a lack of housing, drug and alcohol dependencies, feelings of stigma and shame, and serious physical disabilities, seemed to get in the way of focusing on medication adherence per se.

Although many study participants identified having struggled to obtain stable housing at some point since being diagnosed with HIV, more virally unsuppressed than suppressed participants (10 versus 4) continued to face housing problems even at the end of the study. They described having to keep their medications in a backpack or at a friend’s house when they were living in a shelter or on the street. It was sometimes challenging to make or keep clinic appointments when unsure about *“where I would be sleeping”* when the next visit was scheduled. An Atlanta participant described his situation:

*I was looking for housing some time ago and I wasn’t assisted with that, so I’m living, I always say communal living … it’s a single home, but there’s … actually ten different people living in the house. They’ve broken it up into little rooms. Um, the living conditions are extremely poor. There is one refrigerator for ten people to use, the refrigerator does not work. My food that I do have is being stolen all the time. … Um, they’re playing loud music. They’re smoking all kinds of drugs that I can, seeps through the door, I have to put stuff down at the bottom of my door, so I don’t smell them. … I’m not tending to myself in a way that it should be done. It’s one community bathroom. The one woman that uses the bathroom, um I’m gonna try to be as polite as possible, when it is her time of the month, it ends up on the toilet seat and she doesn’t clean it up. … It’s a very, it’s a disgusting, poor living condition, and I can’t take care of myself at all. So, on top of your depression, you go for your food and it’s not there. I’m not eating. So that all leads up to poor health. (49 year-old White participant at the Atlanta site) - SOC arm*.

While some unsuppressed participants had been linked to mental health services before or during the study, about a third of virally unsuppressed intervention and SOC participants (n = 16) described on-going, unresolved struggles with mental health issues, social stigma and substance use that interfered with healthy living and with medication adherence. Some unsuppressed participants struggled with anxiety or depression because of their HIV diagnosis, sorting through issues of disclosure to sexual partners and family, and/or accepting the need to take medications for a lifetime. For others, such mental health issues were precipitated by other life events – the loss of a family member or having to manage other serious health problems. For many, these issues were exacerbated by food, employment, and housing insecurities. Resorting to drugs or alcohol to alleviate the stress also contributed to missed appointments or pills.

*I don’t know if it’s ever easy, you know when you live with this uh, it kind of overshadows, basically your life experience. […] Sometimes, it’s just a little depressing, you’re reminded each day, you know, each day you take the meds, you’re reminded of your condition, or my condition… I don’t think anybody really likes meds, but they’re essential to our well-being, you know. I guess I’m grateful that they do have all these different meds today that can help with different conditions. You know, and I’ve got other medical conditions also, so it’s not just the HIV that I’m dealing with, so. (55 year-old Black participant at the Boston site) - SCM arm*.

Challenges accepting the disease, the need to take HIV medications or deal with their side effects led three participants to discontinue their medications and to leave their fate *“in God’s hands.”* As one participant explained who was diagnosed in 1998 but hadn’t taken HIV drugs since 2011:

*Well, um, it was a choice that I made um, when I was... and they had put me on medication, and it made me real sick. And when I stopped taking the medication, I was – I got back to feeling fine, so you know, I’m kind of superstitious. I prayed about it and put it in the hands of God and […] I guess you can say I’m not, I, I don’t, um. How can I say it? I don’t claim it. I don’t claim the um, disease and I just live day-by-day. And I’ve been alright. I haven’t been sick. I haven’t, have not ever been hospitalized. And I really haven’t had no problems as far as the HIV is concerned, since I stopped taking the medicine. You know, I’ve just been, honky dory so far. (50 year-old Black participant at the Atlanta site) – SOC arm*.

In general, both SCMs and CCMs helped people obtain Social Security disability, deal with lost IDs or other issues. They provided tokens, bus passes and sometime actual *“lifts”* for unsuppressed patients to get to their clinic visits. However, neither group of counselors was always oriented towards assisting with the root problems that virally unsuppressed participants faced. This seemed especially the case for CMs in the SOC arm. Only eight of 22 virally unsuppressed SOC participants described receiving any kind of support, and this was generally described as cursory. Several participants in this group described knowing more (from years of dealing with HIV) than their case worker did.

*The one (social worker) that I had before, they don’t do nothing. I don’t know about the one now because I’m gonna start new with him today. But uh, the ones that I have, and they supposed to help with housing and with other things. They never, they never help me with, with nothing. And the, and the time that she see me, it’s because the doctor push her to see me… Yeah, uh, housing, but they don’t, they don’t, but seriously, they can’t help me with my health. Why would they help me with my housing problem? (44 year-old Puerto Rican participant at the Boston site) – SOC arm*.

#### Intervention Challenges from the SCM Perspective

**From the SCMs’ perspectives** one of the main benefits of the intervention was the more personalized care and attention they could provide to patients. One SCM (#1) explained that clinic-based social workers might have 500–600 other patients. Because the SCMs were not employed through the clinics, they could offer their *“undivided attention,”* more flexible scheduling, and no time limit during their sessions. Using a MI approach, SCMs would try *“tapping into what they (patients) thought was important to them, whether that’s their family, or they want to go to school, or just whatever it is to stay healthy.”* By providing a sympathetic and non-judgmental ear, the intervention works to *“build an extra layer of trust.” (SCM #3)*.

*I found what seemed to be most helpful to clients is to have a sympathetic, nonjudgmental ear, aside from their medical team to help them, you know, just put a handle on what was going on with regard to --… Medication adherence and what have you and sometimes that seemed to make a difference, that really you know playing the role of you know um a nonmedical provider, you know I was more uh perceived I think as an ally by them in their efforts to become adherent. (SCM #4)*

Most SCMs acknowledged, however, that the intervention only appeared to work if participants put in effort to engage with it. Echoing statements by a few participants in the SCM arm, several SCMs acknowledged that some participants already had a good support system in place and did not need the additional services, and occasionally a participant might just want to work the system, only making visits that would be reimbursed by the study.

SCMs concurred with the mental health and structural challenges described by participants. They acknowledged that the biggest barriers to medication adherence were often *“life circumstances”* (e.g., housing and job insecurity, mental health and substance use issues) that prevented them from engaging in care. Helping participants navigate these structural barriers required having good working relationships and networks with other providers for services outside of a SCM’s job scope (SCM #1). Linkage to additional services was difficult in at least one study location, because *“things here are very scattered.”* A patient might have to go to multiple places across town to access different services (SCM #2). As SCM #3 noted, a person could be on a housing list for 12 months before lodging became available.

SCMs had mixed views on MI. Although most identified MI techniques as especially suitable to help participants get in touch with their intrinsic desires to better manage their disease and *“get off the fence,”* they also concluded that to be successful MI requires *“a revolution in your thought processes”* (SCM #4) – and not just the participant’s thinking, but also the counselor’s. In different ways, SCMs questioned how well a participant who was *resistant*, *in denial* or facing major life challenges could generate his own plan of action. If MI were to work under these circumstances, they suggested that the intervention dose should be larger – for example, having weekly or at least monthly visits for a longer duration. Although SCMs joined the study with different levels of experience using MI techniques, most suggested that MI was *“a really hard skillset to learn”* (SCM #6), particularly when working with clients in such challenging situations and over such a short period of time. They concluded that the two-day MI training they received at study initiation was not adequate to ensure SCMs possessed the correct mind and skillsets at the beginning of the intervention, rather than building them along the way. Recommendations included conducting a two-week intensive training followed by weekly coaching sessions.

## DISCUSSION

Our qualitative exit-interview study provided greater insight into the main findings from HPTN 078, including an in-depth description of the multiple barriers to adherence faced by this largely “out-of-care” population, as well as a more nuanced understanding of the benefits and challenges of implementing MI.

First, despite SCMs’ questions about how well they were able to implement the MI approach, it was clear that the study CM created a relationship that was different from patients’ experiences in routine clinical care. This emotional bond was noticed, strongly appreciated – and in a few circumstances, its absence after the study closure was regretted. Although SCMs linked people with other services, they did not provide the services themselves, focusing more on the motivational aspects of care and medication adherence. Regardless of viral suppression status, participants in the SCM arm felt acknowledged and supported. On the other hand, peoples’ experiences with CCMs were more variable. Some CCMs were able to create a bond, focusing not just on helping to fill paperwork or schedule appointments, but also showing personal interest and support. Frequently, however, patients experienced gaps in services, including high turnover in staffing, apathetic and sometimes judgmental treatment from support staff.

Although not directly expressed, participants may have valued the SCM relationship (and MI’s client-centered approach) because it contrasted with stigmatizing interactions often experienced or anticipated, particularly by PLWH of color. Indeed, some have suggested that the patient-centered nature of MI may help to reduce the imbalance of power between providers and patients of color, restoring the psychological power of those traditionally disenfranchised (14).

Nevertheless, despite the perceived high quality of the intervention, the main study found no differences in VS. One explanation is that, especially for this hard-to-reach population, it takes time to get to viral suppression, perhaps because they had to sort out so many of the other (individual, social, structural) deterrents to care and medication adherence. Overall, only 48% of study participants were virally suppressed at the end of the study (Remien, under review). Participants who were suppressed, whether in the CM or SOC arm, appeared to have fewer intractable barriers to their care. While they might need assistance with insurance, fewer needed support from their CMs to address issues of housing or food insecurity. In some cases, they had already developed the personal skills and/or access to networks to take care of these needs. Nonetheless, many virally suppressed participants described having struggled with such issues in the past. Many virally unsuppressed participants attributed their challenges with visit and medication adherence to homelessness, housing or food insecurity, mental health and/or substance use problems, other chronic health issues, including blindness, or underlying job and financial security. In some cases, CMs were working with them to resolve these issues; in other cases, the system seemed ill-equipped to address them. A synthesis of qualitative studies exploring underlying reasons for poor treatment outcomes among Black and Latino PLWH identified similar phenomena in which participants’ cyclic and/or long terms experiences of material, social and emotional challenges wore down their sense of self-worth and their ability or resolve to remain engaged in care (25).

Some researchers have found that, while MI leads to subjective improvements in quality of life and reduction in some risk behaviors, this has not necessarily translated into changes in objective measures of adherence or VS (26–28). Furthermore, consistent with the SCM’s experience, there is extensive literature on the difficulty of learning MI (29). This includes not only learning the client-centered components of MI, but also the technical component (the systematic evocation of statements from participants that argue for change or against the status quo and the sidestepping of statements that argue against change), which is considered an important mechanism of action in MI (30). Other study-specific issues, such as the decision to forgo an intervention manual in order to avoid rigidity in the intervention and an unfortunate lengthy gap between the initial MI training and study activation, may have also contributed to the difficulties in the SCMs learning MI to proficiency.

The challenges encountered by our SCMs to learn and deliver MI impeded our ability to accurately assess its efficacy in improving HIV outcomes among HIV virally unsuppressed patients, many of whom had been out-of-care. Nonetheless, the established efficacy of MI to improve HIV, mental health and substance use outcomes in other studies (15–17) suggests that MI could improve outcomes in this challenging population. For example, many virally unsuppressed patients in our study noted the non-judgmental approach used by the SCMs (and not as much by their CCM), which is critical to engaging individuals whose non-adherence to treatment may have led them to experience judgmental interactions with providers in the past. That MI can be used to improve outcomes across a variety of adherence-related issues is also a strength, since it requires the provider to master one counseling intervention, rather than multiple interventions, to address each factor impeding treatment engagement and viral suppression. However, aside from potentially increasing motivation to follow-up with appointments for needed services, MI does not directly address the structural impediments that unsuppressed PLWH may encounter in engaging in care and achieving viral suppression. Future studies considering the use of MI to address HIV treatment adherence among such a challenging population should conduct a rigorous review of how MI has been used in prior studies to hopefully achieve better outcomes. Furthermore, those studies may want to ensure that counselors have been trained to proficiency *before* the outset of study and supported with fidelity monitoring and coaching *throughout* the study to sustain fidelity and allow an accurate assessment of the efficacy of MI (31).

Several limitations should be kept in mind when considering our findings. The study aimed to identify the “hard-to-reach” PLWH; those who had been out of care. Due to challenges with slow recruitment, strategies shifted away from deep-chain, respondent-driven sampling towards a wider range of recruitment strategies, including direct recruitment through clinic advertisements (32). Through the exit interviews, it was clear that some participants in the intervention arm had been or were currently in the care of case managers who were providing support with care and/or medication adherence or linkage to other non-HIV related services – diluting the effect of the intervention. In addition, our efforts to compare participants by VS status are at best a “blunt” instrument. We used the last viral load test to categorize participants. However, in some cases, participants were interviewed a full 10 months after their last study viral load test. Even among participants who were interviewed within several months of their last test, we recognize that VS status is not static and may have changed due not only to adherence behavior, but also viral resistance to medication. Finally, the SCMs were not embedded into a clinical team and providing medical management, whereas the SOC CMs may have had a longer history of interactions with patients in their care and perhaps a more complete knowledge of their medical history.

## Conclusions

This qualitative exit study was conducted among a sample of mostly Black or African American MSM living with HIV, most of whom were out-of-care and all of whom were virally unsuppressed when initially enrolled in a randomized trial to assess the effect of an enhanced case management intervention on viral suppression. Our findings reinforce the literature suggesting the benefits of an MI approach to support PLWH intrinsic motivation for medication adherence, provide social support and counter stigma. However, they also provide a more in-depth picture of the multiple systemic challenges, including housing and job insecurity, mental health, substance use and other chronic health issues – barriers that may not be easily resolved through MI alone. Our findings suggest that an enhanced case management approach, one that includes the rapport-building qualities of MI, can have very positive effects on PLWH attitudes towards medication adherence, but that more intensive and coordinated support to address the broader structural barriers is required for some, not all, of those who have fallen out of care or remain virally unsuppressed. A differentiated care model that provides a more intensive MI plus structural intervention approach for patients facing multiple systemic challenges warrants further evaluation.

## Electronic Supplementary Material

Below is the link to the electronic supplementary material.

Supplementary Material 1

## Data Availability

Requests in writing to HPTN leadership to use de-identified qualitative data from exit interviews will be considered on a case-by-case basis.
