# Supplementary material for: “The role of case management in HIV treatment adherence: HPTN 078”
Source: AIDS Behav. 2022 Apr 1;26(9):3119–30. doi: 10.1007/s10461-022-03644-2 (PMC9371990; doi:10.1007/s10461-022-03644-2)
Supplement: Supplementary file 1 — Supplementary Material 1 [file 10461_2022_3644_MOESM1_ESM.docx]

Table 2. Exemplary quotes, by study arm and viral suppression status, of general experiences with case managers and specific types of support provided. ***Bolded text added for emphasis.***

| **TOPIC AND VS STATUS** | **CM INTERVENTION** | **SOC ARM** |
| --- | --- | --- |
| **POSITIVE TREATMENT** | **Like a friend…** | **Dedicated professional…** |
| Suppressed | Participant: *I found (SCM) very easy to work with.  He did not, he was not judgmental, […] if he called for any appointments, he would reschedule, because I missed a few with him.  He was always polite about it, you know.  He never got mad at me, he never yelled at me, you know. … um, and you know,* **he seemed like I was talking to a friend or something***, you know, he made it very easy.  I didn’t feel like I had to hide anything from him.*  *(57 year-old White participant, Boston)* | Participant: *Um, um, it's been – he's very on top of things, like my age and my, my [inaudible 0:39:10.7] everything, um,* ***he is really on top of it.***  *And, and the boring thing is* ***the updates and whatnot, all that paperwork that um, the government um, needs*** *and whatnot. He, he is very um, he makes it comfortable for you, because he'll, he'll, she won't tell – he'll tell you what time, like hey, in such and such month. Um, why won't you put in a calendar that such-and-such month we're gonna um, be doing this, or we're going to do this again and whatnot sometime around this time.*  *(48 year-old Black participant, Boston)* |
| Unsuppressed | Participant: *She was like fresh air for me. Um, she said the open and just so easy to talk to and understand me. … it’s like I’m out of at the clinic,* ***because it’s like I’m talking to a friend, or a best friend, or a cousin****, and somebody that they’ll go see, I can just really, just can be. So, she made it like very, very easy to talk to her. Just made me very comfortable with speaking about my HIV status, which may, you know, want to be more comfortable and more diligent in taking my medicine.*  *(31 year-old Black participant, Birmingham)* | Participant: *It was just comfortable talking to him. Because he made it myself, like I told him what first bring me in what was going on, so he made it a priority to make sure that I do the right thing, you know what I’m saying.* ***Maybe I needed somebody a little stronger than I am in my eyes, he is stronger than I am****. You need to do this, you need to do that, whatever. And didn’t sugar coat it.*  *(41 year-old Black participant, Atlanta)* |
| **NEUTRAL TREATMENT** | **Good but no real need** | **Polite but no real action** |
| Suppressed | Participant: *That's why a lot – this is why I think about these questions that you're asking me from these questions require um, are kind of like, something I required or did I need it, which I really didn't. So, and especially from certain areas of my care…* ***I have no complaints. No nothing bad to say****. Honestly, everything I’ve done, I think it was, I think it was a wonderful program, study. (50 year-old Black participant, Baltimore)* | Interviewer: Okay. So, you haven’t really had any interaction with him yet*?*  Participant: ***No, but he’s just polite on the phone and he’s attended to my needs just like the rest of them*** *and it must rain down somewhere, I don’t know. (51 year-old multi-racial participant, Boston)* |
|  | **Med support but less continuity** | **Med support but less continuity** |
| Unsuppressed | Participant: *Well, you know, like I said the level of care is in my opinion deteriorated over the years. I mean,* ***I'm still on the same meds and whatnot, but you know, just the fact that I'm seeing someone different having to go through getting to know that person, trust that person****, that just, you know, too many other factors that weigh in that, you know, I'd prefer not to even deal with….The only thing that I can say in regard to that is this, they do damn good at giving out prescriptions, and they do damn good at writing different prescriptions. As far as like care goes when you throw the word care in there. [Chuckle] It's an interesting place to put that word. You know? Quite a few adjectives I could throw in there, but that's a pretty interesting place where you put that.*  *(57 year-old Black participant, Boston)* | Participant: *I talk to a lot of social workers, because I mean****, my, my social workers might tell me, “No, you can't. I can't help you,”*** *and I can go to another one, or another source and they can help me. So, I mean, sometimes it's, you know, it's just, I like the, the people that take good care of me. But when it's time for me to ask persons for me to try to help myself, I mean, my social worker sometimes put me on the back burner and I really don't like that.*  *(53 year-old Black participant, Birmingham)* |
| **UNSATISFACTORY** | **A few concerns noted** | **CM not very engaged** |
| Suppressed | Participant: *So um, I don't, I don’t – it’s, it’s, it’s a 50/50. I mean, the,* ***the only time I've actually just been put down is getting here and no one responding and then not getting my apartment,*** *but that's never – I mean, I, not getting – it wasn't not getting apartment, getting assistance, but it's, it's still perfectly fine.*  *(23 year-old Black participant, Atlanta)*  (Only one of two comments) | Participant: *But just say that it’s okay every now and then and every once* ***every two or three weeks in between appointments, that you can pick up the phone and say, “How you doing? Um we haven’t heard from you. We just wanna make sure that things are well.*** *Anything we can do? Are there services we can offer?” Um so just you know during this point, sometime your finance may get you know -- and I understand they offer services about shelters sometimes if you need a shelter, if you’re needing you know um transportation and stuff of that nature to get back and forth to the appointments, but so far so good. I am independent you know with transportation. Yeah.*  *(55 year-old Black participant, Birmingham)* |
| Unsuppressed | Participant: *I have a little disagreement with them. I don’t like the person… You know, I’m a human being.* ***You left me waiting in that hall for, for 30 minute and when I sit down you want to use your phone. No, bro, you’re really confused.*** *I come in from far away for coming and sitting down here with you. I’m thinking that you wanna do something important and you coming with that thing to try to play with your phone and the other thing. And I just tell him right away that I don’t like it.*  *(44 year-old Puerto Rican participant, Boston)* | Participant: *I don’t want to make case management sound like it’s not a good thing, because it certainly is.* ***It was just unfortunate the case manager I had was not thrilled with their job or didn’t know enough about their job,*** *so it’s kind of conflicting to me with the answers that I’m giving you, because case management, in my eyes is very, very important, and I fell into an unfortunate situation where I had one that did not want to be part of it.*  *(49 year-old White participant, Atlanta)* |
| **NO RELATIONSHIP** | **Already in care… no need** | **Frequent turnover** |
| Suppressed | Participant: *Well, I’ve already had a clinic. But he tried to tell me something that uh, how he can set all for me and this was it, was just the refills.* ***It wasn’t no more than that, just the refills, because I already have a clinic I go to. And I use their service****.*  Interviewer: Okay. Okay. Did she – did [De-identified] help you with um, other services outside of HIV?  *No. (50 year-old Black participant, Baltimore)* | Participant: *Yeah, well, like I said, that’s not – that’s the only kind of frustrating thing that uh, I, I really went through is that once I would get to know one social worker, then all of a sudden, uh, uh, well, uh, I’m having to have another social worker because uh, she had moved on to something else, he had moved on to something else. And uh, and one time in, within uh,* ***one year, I had six different social workers and uh, I’m like, well, what’s really going on****?*  *(51 year-old Black participant, Birmingham)* |
| Unsuppressed | N/A | Participant: ***I have a social worker, but I don't know what happened to her.***  Interviewer: Okay. How long ago?  Participant: *I’d say years ago I would say, because I’d be located from Richmond to DC and back on DC, back down to Richmond.*  *(49 year-old Black participant, Baltimore)* |
| **HIV-RELATED CARE** | **Helping to recommit to adherence** | **Appointments and paperwork** |
| Suppressed | Participant: *I think that it was talked about.  We never set anything specific, other than, you know,* ***keeping up with my meds, and uh you know, staying clean.****Um, you know he was aware you know, when I relapsed and then he was helpful in encouraging me to keep me on the positive side, you know, so it worked out.  It was good all the way around. …When I did relapse, it wasn’t, he didn’t shun me or yell at me or anything, you know, just work with it.  You know, like it was just another everyday thing, you know.*  *(57 year-old White participant, Boston)* | Participant: *Well, she makes sure that -- if I haven’t made my appointment,* ***she makes sure that appointment gets made*** *-- Unbeknownst to me. Like she’ll just make the appointment, and then they’ll --… Um no, well, she calls me and make -- like she checks in and asks me, “You’re still taking your uh meds. Right?” I be like, “Yeah.” But that’s about it and just checking in to make sure, but nobody has to tell me to take my meds.*  *(34 year-old Black participant, Baltimore)* |
|  | **Helping to commit to adherence** | **Just doing her job** |
| Unsuppressed | Participant: *“You never want to miss a dose!” Once I came back, they were very excited that they got back in touch with me. They both were like – I don’t know, like, when [De-identified] got my phone number, she was – she was like, “Oh my God! I’m so excited to hear from you!” She was like, “I thought you had just, like, left us.” I said, “No, I’m still here.” And* ***I told her what was going on with me personally and everything, and she was like, “Well, I’m glad,” she was like, you know, chasing me****, “[De-identified] needs to see you. I’m going to give him your phone number,” I started talking to [De-identified], we started off with an interview and we went from there.*  *(28 year-old Black participant, Baltimore)* | Participant: *I was at the doctor office to not just to pick up my medicine, and she heard my voice and um, I had missed the doctor appointment like I had told you, and they needed some layout. So she heard my voice and she ran out and she’s, “I’m not gonna let you leave, because we need some labs.” And I said okay, you know, no problem. But I mean,* ***it's things like the social worker just (getting) her job done, so the doctor can get his job done****. But when I, I need you to help me, you know, to get something for me, it don't seem like it, it work like, like it work. It don't really work that way for me.*  *(53 year-old Black participant, Birmingham)* |
| **NON-HIV SERVICES** | **Few additional services needed** | **Few additional services needed** |
| Suppressed | Participant: *I did have a instance where I did ask him about, um, housing. And he told me he could help me, and he’ll get back to me with it, but, um, he really didn’t really help… But, um, at the time, I was, um, I was more focused and driven on, you know, just making sure that I’m okay in the time being. Meaning that, um, I was just trying to make sure that I was, uh, right to go.* ***Okay, well, if nobody else is going to help me, you know, I can definitely make sure I can help myself****…*  *(23 year-old Black participant, Atlanta)* | Participant: *I mean, I do know that there are service – they can refer me to other places where I can get help, like if I needed legal help, or if I – if I actually found myself in a situation where like, I do have a roof over my head and needed to get a place to eat, they know they can point me to places. But um,* ***I've been very lucky and not needed a lot of those extra services****, and that the only thing I really needed to talk to my caseworker on has been about insurance. I’m pretty much a very lucky.*  *(44 year-old Black participant, Boston)* |
|  | **Mental health, housing or other services in process** | **Mental health, housing or other services needed** |
| Unsuppressed | Participant: *I was probably somewhere using drugs.*  Interviewer: Okay, and what’s your drug of choice?  Participant: ***Heroin and cocaine****. […]*  Interviewer: How do you feel about (your viral load going up?]  Participant: *It worries me. Coz I’m more susceptible to obviously infection. And um -- and unhealthier medical regimens you know um, and I am doing my best to stop using.* ***I’ve been going to meetings. Uh I have a good support system, and I actually care about my health you know so anything that I can possibly do to better my health, I am trying.*** *It’s not an easy thing to overcome because I’ve been using for over 30 years, so it’s not easy for me but I try and that’s all that […] Once upon a time -- let me tell you something. Once upon a time, I wouldn’t have the time of day to talk to you on the phone. I would tell you get the hell off my line.*  *(47 year-old Black participant, Baltimore)* | Participant: *(Case worker left) We was starting on finding a place to stay because I was homeless at the time. And, he was like helping, getting into certain things and groups and stuff like. With people you can talk to about it. But really, I really never did do the group sessions like that. So,* ***we were on the verge to getting to a lot of things, but you know he mainly talked to me about HIV care*** *and you know make sure I do the right thing and stuff…Because he knew basically a lot of stuff that I was around or whatever, and it wasn’t healthy for me.* ***I don’t want to go to jail, so I didn’t want to have them take me to jail, so basically, I was trying to stay out of trouble****…No. I was just going to say he helped me with a lot of things. Mostly, like food pantries and stuff like, and you know anything I needed or whatever, he just like gave me a way to get into it, you know what I am saying. Talk to people, you know what I’m saying to help me out and stuff like that.*  *(41 year-old Black participant, Atlanta)* |
